# The Top-20 Studies About Anterior Shoulder Instability From an Altmetric Analysis Had Higher Levels of Evidence Than Those From a Traditional Bibliometric Analysis

**DOI:** 10.1016/j.asmr.2024.100974

**Published:** 2024-07-14

**Authors:** Liam O’Dwyer, Conor Ledingham, Martin S. Davey, Austin Kerin, Azim Huszar, J. Tristan Cassidy

**Affiliations:** a Graduate Entry Medical School, University of Limerick, Limerick, Ireland; b Department of Trauma & Orthopaedic Surgery, University Hospital Limerick, Limerick, Ireland

## Abstract

**Purpose:**

To compare the characteristics of the top-20 studies about anterior shoulder instability according to the Altmetric Attention Score (AAS) with total citation counts.

**Methods:**

Two separate searches were performed for articles related to anterior shoulder instability. The Altmetric search identified the top-20 articles according to AAS. A bibliometric search using Web of Science identified the top-20 most-cited articles. Altmetric criteria were applied to the bibliometric list and vice versa.

**Results:**

The AAS from the Altmetric list ranged from 44 to 432. The highest AAS from the bibliometric search was 70. One study appeared in both lists. Most online mentions were from X (formerly Twitter). The geographical breakdown of X mentions saw 71 countries appearing in the Altmetric search versus 21 in the bibliometric search. The total citations in the bibliometric list ranged from 91 to 358 versus 0 to 121 for the Altmetric list. The Altmetric top-20 list contained 8 studies that were Level II or higher versus 3 in the bibliometric list.

**Conclusions:**

The top-20 studies according to AAS or citation count are not the same. The top-20 studies by AAS are composed of studies at higher levels of evidence versus the top-20 studies when listed by citation count.

**Clinical Relevance:**

Electronic searches are an important way to access information in the modern world. Different search options generate results according to different parameters and may generate different results for the same query. It is important to understand these differences so that users have a better understanding of where the most clinically useful information can be found, especially regarding medical conditions.

Glenohumeral instability is a common cause of shoulder pain and dysfunction, with anterior shoulder dislocation comprising 95% of all shoulder instability cases.^1^ Treatment may be nonoperative or operative; a number of factors including the risk of potential recurrence (i.e., young age or collision athlete) or the desire to return to a high level of activity often lead to the decision for operative treatment.^2^, ^3^, ^4^

Multiple studies have performed a bibliometric analysis on the topic of shoulder instability.^4^, ^5^, ^6^, ^7^ Citation counts are used to calculate a journal’s impact factor (IF). The IF is one of the leading metrics used by funding agencies, universities, and sometimes policymakers to determine the quality of the journal in which a study is published.^8^ There are, however, flaws with IF and citation analysis. Because the passage of time confers an advantage for overall citation number, citations are subject to bias in several ways, namely self-citation, journal bias, the snowball effect, and country bias.^9^, ^10^, ^11^ Despite citations taking time to accrue, publishing in a high-impact journal is sometimes used as a surrogate measure for many authors with respect to impact and subsequent citation.^12^

The introduction of “altmetrics” in 2011 in an ever-growing world of social media has led to a broader perspective in the evaluation of scientific literature, theoretically capturing an article’s wider reach and being able to objectively quantify an article’s immediate attention and impact.^13^ A variety of Web-based metrics are used to determine an article’s Altmetric Attention Score (AAS).

There appears to be a positive correlation between AAS and citations in the orthopaedic literature,^14^, ^15^, ^16^ although no association was found for AAS and citations in shoulder arthroplasty.^17^ A prior study relating to shoulder instability found a weak association between AAS and Web of Science (Clarivate Analytics) IF.^16^, ^17^, ^18^ The characteristics of the most-cited articles versus the highest-scoring Altmetric articles on shoulder instability are unknown.^13^

The purpose of this study was to compare the characteristics of the top-20 studies about anterior shoulder instability according to the AAS with total citation counts. The hypotheses were as follows: (1) The top-20 studies from each search would not be the same in terms of article content; (2) the level of evidence would vary between the 2 lists; and (3) the 2 lists would have differing Altmetric geographical and demographic breakdowns.

## Methods

### Search Strategy

Two separate literature searches were performed in November 2023. The search was limited to a 10-year period between November 2013 and November 2023. The Altmetric Explorer database was searched using the following PubMed query: ((shoulder instability[Title/Abstract]) OR (“recurrent instability”[Title/Abstract]) OR (shoulder stabilization[Title/Abstract]) OR (shoulder labrum repair[Title/Abstract]) OR (Bankart repair[Title/Abstract]) OR (latarjet[Title/Abstract])) AND (“2013/11/13”[Date - Publication]: “2023/11/13”[Date - Publication]). The results were sorted by AAS (highest first). The titles and abstracts were reviewed for relevance using inclusion and exclusion criteria. Of these studies, the top 20 were selected for analysis. The number of articles included was limited to the top 20 to analyze the outlying articles most suited to the stratification system used by each ranking method.

The Web of Science Core Collection database was searched using the following query: ((((((TS=(shoulder instability)) OR TS=(“recurrent instability”)) OR TS=(shoulder stabilisation)) OR TS=(bankart repair)) OR TS=(shoulder labrum repair)) OR TS=(latarjet)) AND DOP=(2013-11-13/2023-11-13). The results were sorted by citation (highest first). The titles and abstracts were reviewed based on inclusion and exclusion criteria. The top 20 of these articles that met the inclusion criteria were selected for analysis.

The inclusion criteria were as follows: (1) studies published between November 13, 2013, and November 13, 2023, and (2) studies relating to anterior shoulder instability (including diagnosis, treatment options [operative and nonoperative], rehabilitation, and postoperative care). The exclusion criteria were as follows: (1) studies with no available English-language translation and (2) studies exclusively relating to posterior instability, arthroplasty, rotator cuff tears, or SLAP tears.

### Analysis

The following bibliometric details were recorded: (1) total citations, (2) citation density, (3) authors, (4) title, (5) year of publication, (6) source journal of article, (7) article type (therapeutic study, prognostic study, diagnostic study, economic study, or controlled laboratory study), (8) article subtype (e.g., case series, cohort study, case-control study, randomized controlled trial [RCT], systematic review, meta-analysis, or review), and (9) level of evidence for clinical research articles. The IF of each journal in the results list was also recorded. The Altmetric details recorded were (1) AAS, (2) Altmetric source category, (3) geographical breakdown, (4) demographic breakdown, and (5) mentions per journal.

### Metric Calculation

The AAS is based on 3 main factors: (1) volume, in which the more mentions there are, the higher the score, and only 1 mention from each person per source is counted; (2) sources, wherein each category of mention contributes a base amount; and (3) authors, in terms of how often the authors mention scholarly content, bias, and who the audience is. How each source is weighted can be seen in Table 1. The AAS was automatically calculated by Altmetric (www.altmetric.com). Citation density was calculated by dividing total citations by number of years since publication. The level of evidence was determined using guidelines published by *The Journal of Bone and Joint Surgery*.^19^ Analysis was conducted using a combination of the Altmetric Explorer and Web of Science databases.

**Table 1:** AAS Source Category Weighting

Altmetric Source Category	AAS Weighting
News	8
Blog	5
Policy document (per source); patent; Wikipedia	3
Peer review (Publons, Pubpeer); Weibo (not trackable since 2019 but historical data are kept); Google+ (not trackable since 2019 but historical data are kept); F1000; syllabi (Open Syllabus)	1
LinkedIn (not trackable since 2014 but historical data are kept)	0.5
X (formerly Twitter; posts and re-posts); Facebook (only a curated list of public pages); Reddit; Q&A (Stack Exchange); YouTube	0.25

AAS, Altmetric Attention Score.

## Results

### Altmetric Explorer Database Search

**Fig 1:**
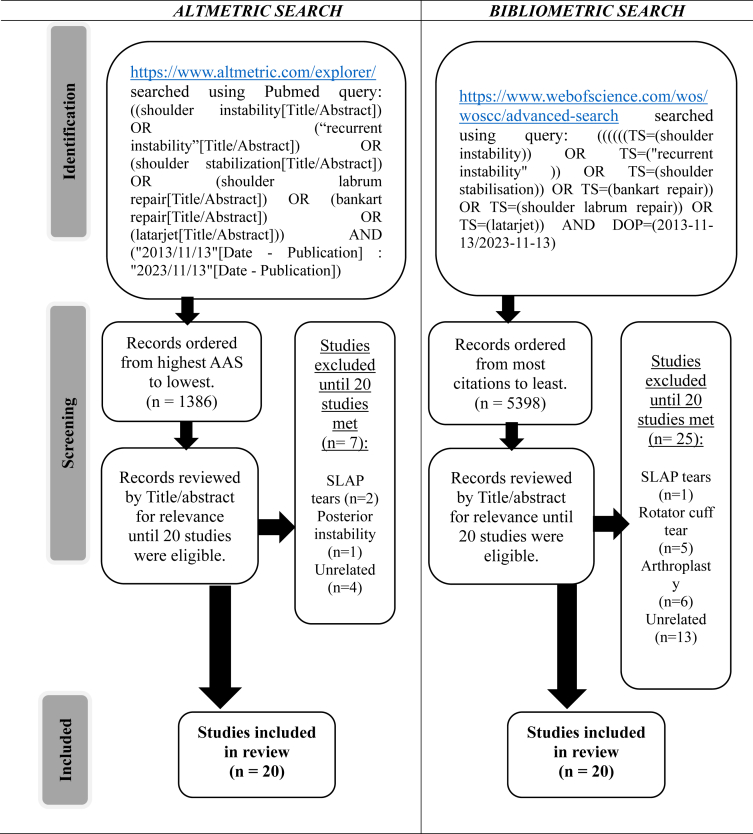
Flowchart of search strategies for Altmetric search and bibliometric search. (AAS, Altmetric Attention Score.)

**Fig 2:**
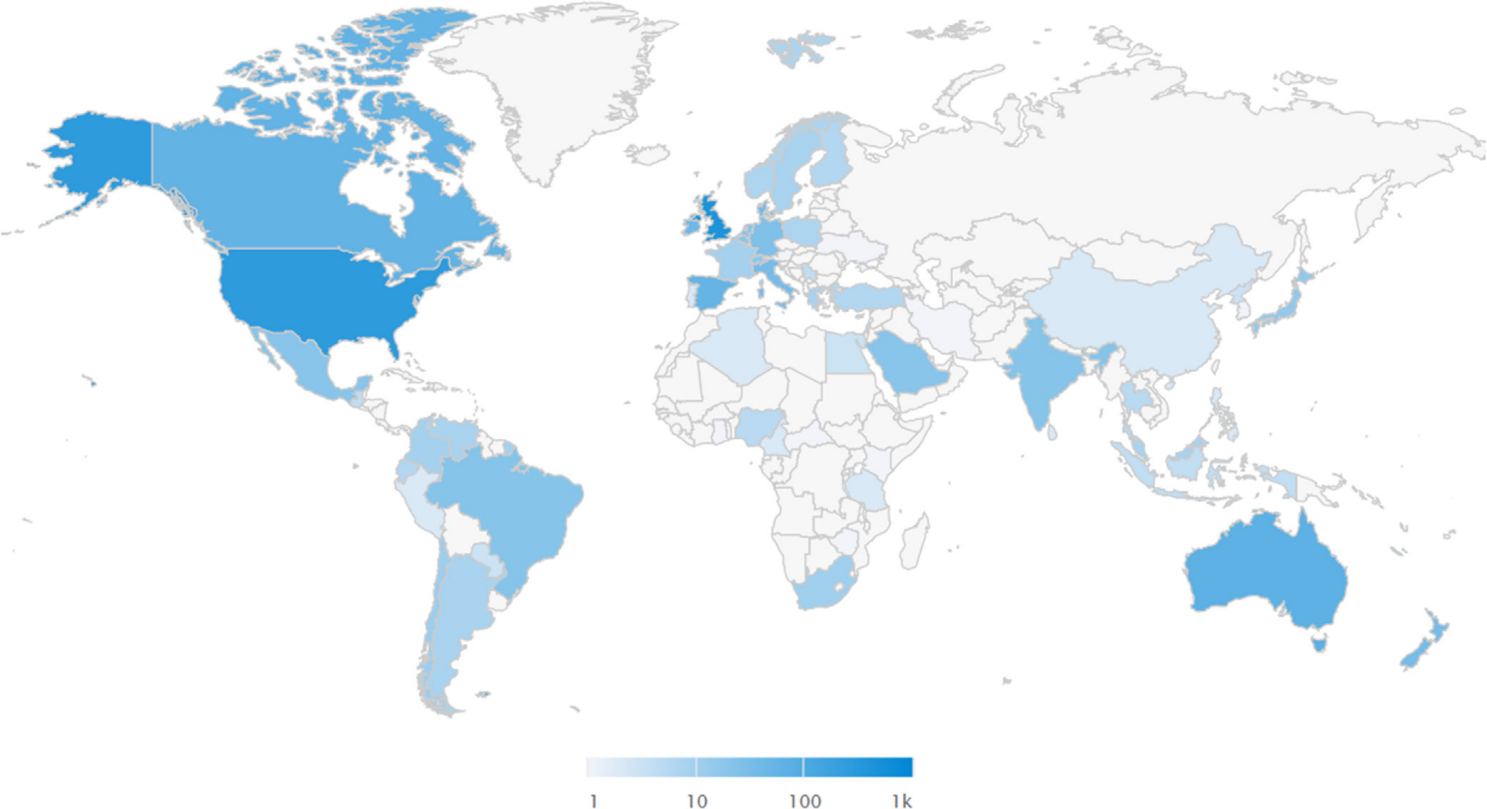
Map of geographic distribution of X mentions from top-20 Altmetric list.

The study identification process is illustrated in Figure 1. This resulted in 1,386 research outputs, of which 1,251 have been mentioned. The top-20 studies according to the AAS can be seen in Table 2. The highest AAS was 432,^20^ with the lowest being 44.^21^ The combined AAS was 1,866, with a mean of 93.3 ± 83.4. The AAS breakdown included 2,216 mentions on X (formerly Twitter), 107 on Facebook (Meta), 76 on news, 8 on blogs, 4 on Wikipedia (Wikimedia Foundation), and 1 on Google+ (Google). Members of the public contributed to the most X mentions, with 1,343 mentions (72.5%). This was followed by practitioners (270, 14.6%), scientists (209, 11.3%), and science communicators (28, 1.5%), with 2 classified as unknown (0.1%). The geographical breakdown of X mentions showed that 1,406 unique X users had posted about this content in 71 countries; a representation of this can be seen in the map in Figure 2. The United Kingdom had the most mentions, with 423 mentions (19.1%) from 225 unique users. The United States was second, with 269 mentions (12.1%) from 194 unique users. The country could not be specified for 826 mentions (37.3%) from 528 unique users.

**Table 2:** Top-20 Studies According to AAS

Rank	AAS	Title	Citations (Density)
1	432	“Anatomic and Biomechanical Comparison of the Classic and Congruent-Arc Techniques of the Latarjet Procedure” (2017)	42 (6.0)
2	160	“Treatment After Traumatic Shoulder Dislocation: A Systematic Review With a Network Meta-analysis” (2018)	19 (3.2)
3	150	“Short-term Effectiveness of High-Load Compared With Low-Load Strengthening Exercise on Self-Reported Function in Patients With Hypermobile Shoulders: A Randomised Controlled Trial” (2022)	4 (2.0)
4	94	“Motor Control Exercises Compared to Strengthening Exercises for Upper- and Lower-Extremity Musculoskeletal Disorders: A Systematic Review With Meta-analyses of Randomized Controlled Trials” (2021)	9 (3.0)
5	92	“Anterior Shoulder Instability in Throwers and Overhead Athletes: Long-term Outcomes in a Geographic Cohort” (2022)	5 (2.5)
6	85	“Quantitative Assessment of the Coracoacromial and the Coracoclavicular Ligaments With 3-Dimensional Mapping of the Coracoid Process Anatomy: A Cadaveric Study of Surgically Relevant Structures” (2018)	26 (4.3)
7	80	“Arthroscopic Capsular Shift Surgery in Patients With Atraumatic Shoulder Joint Instability: A Randomised, Placebo-Controlled Trial” (2023)	0
8	75	“Heavy Shoulder Strengthening Exercise in People With Hypermobility Spectrum Disorder (HSD) and Long-Lasting Shoulder Symptoms: A Feasibility Study” (2020)	15 (3.8)
9	72	“Rotator Cuff Muscle Imbalance Associates With Shoulder Instability Direction” (2023)	2 (2.0)
10	72	“Arthroscopic Bankart Repair Versus Conservative Management for First-Time Traumatic Anterior Shoulder Instability: A Systematic Review and Meta-analysis” (2020)	51 (12.8)
11	71	“BESS/BOA Patient Care Pathways: Atraumatic Shoulder Instability” (2019)	0
12	70	“Who Will Redislocate His/Her Shoulder? Predicting Recurrent Instability Following a First Traumatic Anterior Shoulder Dislocation” (2019)	19 (3.8)
13	70	“Risk Factors Which Predispose First-Time Traumatic Anterior Shoulder Dislocations to Recurrent Instability in Adults: A Systematic Review and Meta-analysis” (2015)	125 (13.9)
14	52	“Prospective Evaluation of Glenoid Bone Loss After First-time and Recurrent Anterior Glenohumeral Instability Events” (2019)	55 (11.0)
15	51	“Recurrence After Arthroscopic Labral Repair for Traumatic Anterior Instability in Adolescent Rugby and Contact Athlete” (2018)	55 (9.2)
16	51	“A Clinical Review of Return-to-Play Considerations After Anterior Shoulder Dislocation” (2016)	35 (4.4)
17	49	“Return to Sport After Surgical Treatment for Anterior Shoulder Instability: A Systematic Review” (2018)	60 (10.0)
18	48	“Neuromuscular Exercises Improve Shoulder Function More Than Standard Care Exercises in Patients With a Traumatic Anterior Shoulder Dislocation: A Randomized Controlled Trial” (2020)	17 (4.3)
19	48	“Effectiveness of Combined Surgical and Exercise-Based Interventions Following Primary Traumatic Anterior Shoulder Dislocation: A Systematic Review and Meta-analysis” (2023)	1 (1.0)
20	44	“A Functional Magnetic Resonance Imaging Study of Patients With Polar Type II/III Complex Shoulder Instability” (2019)	5 (1.0)

AAS, Altmetric Attention Score.

The top-20 studies were cited 545 times, with the highest number of citations being 125.^22^ Two studies received 0 citations.^23^^,^^24^ The highest citation density was 13.9,^25^ and the mean citation density was 4.9 ± 4.2. There were 9 therapeutic studies, 8 prognostic studies, and 3 basic science studies. The most frequently occurring article subtype was a systematic review, with 5 such studies appearing; this was followed by RCT and cohort studies, with 3 each. Case series and reviews appeared twice each, whereas cross-sectional studies, case-control studies, and meta-analyses were represented once each. Of the 17 studies for which the level of evidence could be assigned, 2 were Level I, 6 were Level II, 3 were Level III, 5 were Level IV, and 1 was Level V (Table 3).

**Table 3:** Number of Studies at Each LOE From Bibliometric and Altmetric Top-20 Lists

LOE	Bibliometric List	Altmetric List
I	1	2
II	2	6
III	8	3
IV	7	5
V	1	1

LOE, level of evidence.

*The American Journal of Sports Medicine* and *British Journal of Sports Medicine* (BJSM) were the two most frequently occurring journals in this list, both appearing five times each. There were 11 journals appearing in total these can be seen in Table 4. The journal with the highest IF was BJSM, with 18.6, whereas the journal with the lowest IF was *Shoulder & Elbow*, with 1.3. The oldest study in this list was from 2015^22^; there were 4 studies published in 2023. The years of publication and the frequency at which they occurred in this list can be seen in Table 5.

**Table 4:** Journals Appearing in Both Top-20 Lists, Along With IF and Total Altmetric Mentions Attributed to Each Journal

Journal Title	IF	Altmetric Top-20 List		Bibliometric Top-20 List	
		Studies	Altmetric Mentions	Studies	Altmetric Mentions
*British Journal of Sports Medicine*	18.6	5	898	1	116
*The Journal of Bone and Joint Surgery—*American Volume	5.3			2	8
*BMJ Open Sport & Exercise Medicine*	5.0	1	135		
*The American Journal of Sports Medicine*	4.8	5	323	7	76
*Arthroscopy*	4.7	2	250	4	31
*Scientific Reports*	4.6	1	95		
*Clinical Orthopaedics and Related Research*	4.3			2	4
*Physical Therapy*	3.8	1	182		
*Sports Health*	3.3	1	52		
*Journal of Shoulder and Elbow Surgery*	3.0	1	116	2	9
*Orthopaedic Journal of Sports Medicine*	2.6	1	96		
*Pilot and Feasibility Studies*	1.7	1	136		
*Shoulder & Elbow*	1.3	1	129		

IF, impact factor.

**Table 5:** Number of Studies Published Each Year From Both Bibliometric and Altmetric Top-20 Lists

Year of Publication	Bibliometric List	Altmetric List
2013		
2014	9	
2015	4	1
2016	4	1
2017	3	1
2018		4
2019		4
2020		3
2021		1
2022		2
2023		3

### Web of Science Bibliometric Search

The study identification process is illustrated in Figure 1. After an initial return of 5,398 results, the top-20 studies according to total citations can be seen in Table 6. The most-cited study in this list had 358 citations^26^; the least cited study had 91 citations.^27^ The studies were cited a total of 2,866 times, with a mean of 143.3 ± 69.6. The highest citation density was 35.8,^26^ and the mean was 16.0 ± 7.0. There were 16 therapeutic studies, 3 prognostic studies, and 1 basic science study. The 19 therapeutic and prognostic studies comprised 6 cohort studies, 5 case series, 2 meta-analyses, 2 systematic reviews, 2 case-control studies, 1 RCT, and 1 review. Of the 19 studies for which the level of evidence could be assigned, 1 was Level I, 2 were Level II, 8 were Level III, 7 were Level IV, and 1 was Level V (Table 3).

**Table 6:** Twenty Most-Cited Articles Pertaining to Shoulder Instability

Rank	Citations (Density)	Title	AAS
1	358 (35.8)	“Evolving Concept of Bipolar Bone Loss and the Hill-Sachs Lesion: From Engaging/Non-engaging Lesion to On-Track/Off-Track Lesion” (2014)	22
2	305 (33.9)	“Redefining Critical Bone Loss in Shoulder Instability: Functional Outcomes Worsen With Subcritical Bone Loss” (2015)	2
3	220 (22.0)	“Long-Term Results of the Latarjet Procedure for Anterior Instability of the Shoulder” (2014)	3
4	158 (19.8)	“Long-Term Restoration of Anterior Shoulder Stability: A Retrospective Analysis of Arthroscopic Bankart Repair Versus Open Latarjet Procedure” (2016)	12
5	153 (15.3)	“The Open Latarjet Procedure Is More Reliable in Terms of Shoulder Stability Than Arthroscopic Bankart Repair” (2014)	2
6	136 (13.6)	“Epidemiology of Primary Anterior Shoulder Dislocation Requiring Closed Reduction in Ontario, Canada” (2014)	11
7	134 (13.4)	“Latarjet, Bristow, and Eden-Hybinette Procedures for Anterior Shoulder Dislocation: Systematic Review and Quantitative Synthesis of the Literature” (2014)	1
8	128 (14.2)	“A Proficiency-Based Progression Training Curriculum Coupled With a Model Simulator Results in the Acquisition of a Superior Arthroscopic Bankart Skill Set” (2015)	16
9	125 (13.9)	“Risk Factors Which Predispose First-Time Traumatic Anterior Shoulder Dislocations to Recurrent Instability in Adults: A Systematic Review and Meta-analysis” (2015)	70
10	122 (15.3)	“A Systematic Review and Meta-analysis of Clinical and Patient-Reported Outcomes Following Two Procedures for Recurrent Traumatic Anterior Instability of the Shoulder: Latarjet Procedure vs. Bankart Repair” (2016)	2
11	117 (16.7)	“Critical Value of Anterior Glenoid Bone Loss That Leads to Recurrent Glenohumeral Instability After Arthroscopic Bankart Repair” (2017)	0
12	111 (11.1)	“The Outcomes and Surgical Techniques of the Latarjet Procedure” (2014)	2
13	108 (15.4)	“Results of Arthroscopic Bankart Repair for Anterior-Inferior Shoulder Instability at 13-Year Follow-Up” (2017)	12
14	107 (10.7)	“The Arthroscopic Latarjet Procedure for Anterior Shoulder Instability 5-Year Minimum Follow-Up” (2014)	1
15	106 (13.3)	“A Guided Surgical Approach and Novel Fixation Method for Arthroscopic Latarjet” (2016)	0
16	103 (10.3)	“Arthroscopic Bristow-Latarjet Combined With Bankart Repair Restores Shoulder Stability in Patients With Glenoid Bone Loss” (2014)	2
17	99 (12.4)	“Clinical Validation of the Glenoid Track Concept in Anterior Glenohumeral Instability” (2016)	1
18	93 (9.3)	“Return to Play and Recurrent Instability After In-Season Anterior Shoulder Instability: A Prospective Multicenter Study” (2014)	43
19	92 (13.1)	“The Effect of Subcritical Bone Loss and Exposure on Recurrent Instability After Arthroscopic Bankart Repair in Intercollegiate American Football” (2017)	17
20	91 (10.1)	“The Effect of a Combined Glenoid and Hill-Sachs Defect on Glenohumeral Stability: A Biomechanical Cadaveric Study Using 3-Dimensional Modeling of 142 Patients” (2015)	4

AAS, Altmetric Attention Score.

A total of 6 different journals appeared in this list. *The American Journal of Sports Medicine* appeared most frequently (8 times). The second most frequent journal was *Arthroscopy* (6 times), followed by the *Journal of Shoulder and Elbow Surgery* (3 times). The journal with the highest IF on this list was BJSM, with 18.6, whereas the journal with the lowest IF was the *Journal of Shoulder and Elbow Surgery*, with 3.0 (Table 4). The year of publication ranged from 2014 to 2017. Nine studies in total were published in 2014 (Table 5).

**Fig 3:**
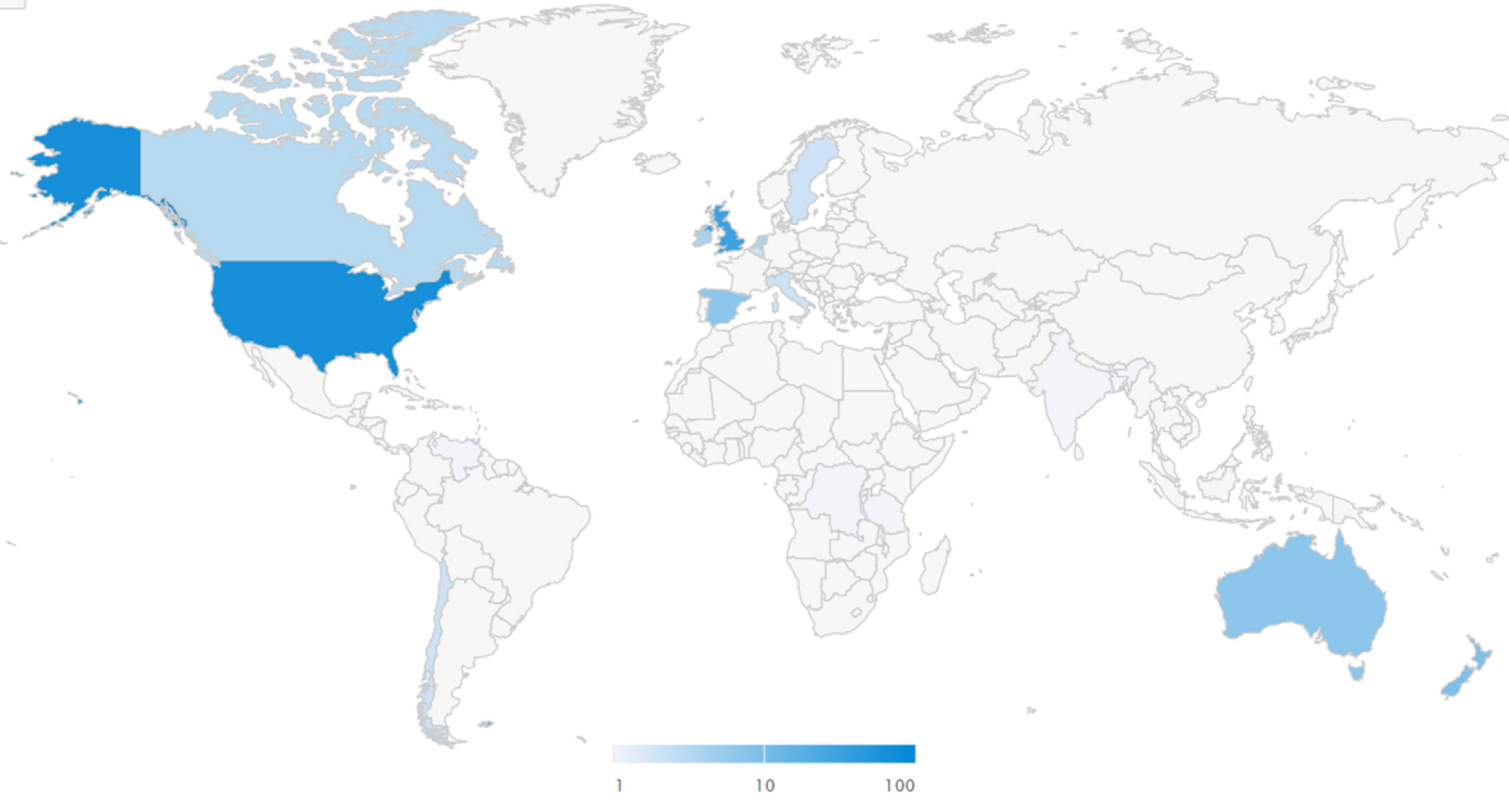
Map of geographic distribution of X mentions from top-20 bibliometric list.

The highest AAS was 70^22^; the study receiving this score was the only study to appear in both searches. The total AAS for the top-20 studies was 223, with a mean of 11.2 ± 17.0. Of the 244 mentions, most came from X (194, 79.5%) (Fig 3). The geographical breakdown of these X mentions revealed mentions from 21 different countries. The United States contributed the most mentions, with 65 mentions (33.5%) from 52 unique users (33.1%), whereas the United Kingdom was in second place, with 31 mentions (16%) from 22 unique users (14%). There were 48 mentions (24.7%) from 42 unique users (26.8%) for which the location was labeled as “unknown” or “other.” The demographic breakdown revealed that most of the mentions were from members of the public (114, 66.3%), with practitioners in second place (37, 21.5%). Scientists accounted for 16 mentions (9.3%) and the scientific community contributed 4 mentions (2.3%), whereas 1 mention was classified as unknown.

## Discussion

The findings of this study confirm that the top-20 list from each search was not the same regarding article content, the Altmetric top-20 list contained more high-quality studies than the bibliometric top-20 list, and the 2 lists had contrasting Altmetric geographically and demographic breakdowns. The study with the highest AAS performed an anatomic and biomechanical comparison of the classic and congruent-arc techniques of the Latarjet procedure.^20^ The Latarjet procedure was labeled a “hot topic” in 2014,^28^ and this is reflected in the bibliometric list, with 6 of the 9 studies from 2014 relating to the Latarjet procedure. The studies in third place^29^ and fourth place^30^ on the Altmetric list focused on nonoperative management of shoulder instability. Excluding the article with the highest AAS, there was a difference in the content of the articles among the top 5 in the Altmetric list versus the bibliometric list. The bibliometric list contained much more technical articles relating to glenoid bone loss,^26^^,^^31^ focusing on the long-term outcomes of the Latarjet procedure,^32^ and comparing the open Latarjet procedure with arthroscopic Bankart repair.^33^^,^^34^ The Altmetric studies, in contrast, focused on exercises for instability,^29^^,^^30^ presented a review of treatment options,^25^ and focused on outcomes in an athletic population.^35^ Therefore, it could be construed that these studies may be more relevant to the patient population in terms of informed patient decision making, rather than what type of surgical technique to use. It is interesting to note that only 1 study appeared on both lists.^22^ Although not addressed in this study, we believe that this suggests that perhaps the time factor is of major importance, with altmetrics being readily available from the time of publication, whereas citations require a great summation of time prior to accumulation.

There was a clear difference in levels of evidence between the Altmetric and bibliometric lists (Table 3). There were 2 Level I RCTs and 6 Level II studies in the Altmetric list, whereas there was 1 Level I RCT and 2 Level II studies in the bibliometric list. Journals are currently pushing toward high-quality studies, with most journals requiring the level of evidence to be assigned prior to submission. However, as is evident by the bibliometric search, there was a paucity of high-quality studies to gain citation dominance over the past decade, whereas the Altmetric search contained more studies at a higher level of evidence. A bibliometric study relating to osteoarthritis found most of the studies in its top-50 list to be Level II or greater; however, most of these studies were published between 1998 and 2008.^36^ It is interesting to note that our finding of the Altmetric search identifying more high-quality studies is in keeping with the results of a study that showed a positive correlation between the AAS and greater levels of evidence.^37^

As is evident from Table 4, the bibliometric list did not include a journal with an IF of less than 3.0. The Altmetric list, however, contained 3 journals ranked lower than this: *Orthopaedic Journal of Sports Medicine*, *Pilot and Feasibility Studies*, and *Shoulder & Elbow*. The Altmetric list also contained a variety of journals, with 11 versus 6 in the bibliometric list. A flaw with the IF can be attributed to the Matthew effect because part of the prestige component can play a role in citation numbers.^38^ Articles published in journals with higher prestige may receive more citations owing to this preconceived prestige. Additionally, it should be noted that at least 1 of these journals represents a relatively “new” journal, with print having started as recently as 2020. The AAS may help identify articles that may be influential within an area, despite being published in a journal that is deemed less “prominent” if journals are to be considered by IF alone. However, the AAS does not discriminate given that the most frequently occurring journal in the Altmetric list is BJSM, which has by far the highest IF and is a leader in the field.

With time being a positive predictor of bibliometric scoring, as is evident in Table 3, there are clear differences in the dates of publication in the 2 lists. Even though both searches were limited to publications from the past decade, publications in the bibliometric list can be confined to the period of 2014 to 2017, with 9 of these studies coming from 2014 alone. As alluded to previously, time has a positive effect on citations: As time passes, influential articles will accrue citations. Therefore, it would not be possible for newer studies to make this bibliometric list.^5^ To account for this, citation density is calculated in traditional bibliometric searches. When one is performing an analysis of the most influential studies in an area, it may be beneficial, going forward, to factor in the study with the highest AAS. As mentioned previously, IF and, therefore, citations are often used to influence policy and funding. The AAS may be a potential metric to help influence decision making around funding, as well as policy, and identifying areas of potential research.

The geographical distribution of X mentions can be seen in Figures 2 and 3. There is a clear difference between the distributions of the Altmetric list and bibliometric list. As is evident, Africa, Asia, South America, and Central America have very few X mentions (Fig 3), with 7 mentions from 6 countries, representing just 3.6% of mentions. In contrast, as shown in Table 3, it appears that the aforementioned geographical regions represented 10.1% of total X mentions. Previous studies have found a positive correlation between funding and citation impact,^39^ with many countries within these regions potentially lacking institutional funding to establish centers of research excellence and perform prospective studies of robust methodology, as well as lacking the potential to assess open-access publishing. It is reasonable to assume that this may potentially contribute to citations—or a lack thereof. Therefore, perhaps Altmetric mentions may be an alternative measure when assessing what studies are influencing practice in some lower-income countries, with less focus on economic inequalities. Additionally, exposure to a potentially diverse range of research ideas from a variety of cultures may play a role in ensuring patients are well informed.^40^^,^^41^

Citation analysis provides us with the studies that are currently influential within the scientific community. However, it appears that Altmetric provides us with a wider understanding of how influential a study is, as well as the types of studies that are influential depending on the audience. Members of the public and other practitioners and academics involved in different areas cannot contribute to a study’s citations and thus make no contribution to the IF of the journal in question. Furthermore, Altmetric does not lean on industrial or economic influences, whereas it could be argued that this is indeed the case for journals that only allow open-access publishing. However, we can begin to calculate how influential studies are on the aforementioned groups by using the AAS. Altmetric provides a demographic breakdown of persons who have mentioned the study on X and other platforms. If we consider, for instance, X mentions, which was by far the most common type of mention in our study, this shows that many members of the public engaged with this content. The AAS could be a means of quantifying public interest in an area, which will serve to make funding applications more attractive.

### Limitations

This study is not without limitations. It is possible that the Altmetric Explorer database may not have included all articles related to anterior shoulder instability. Similarly, the bibliometric analysis was performed on data from the Web of Science database, so theoretically, there is potential for some literature related to anterior shoulder instability to have been wrongly excluded by default. The AAS is calculated according to the quantity and quality of online mentions, with the quality of mention source being weighted; however, Altmetric does not provide specific details on how this weighting is calculated. Furthermore, the AAS could potentially be subject to manipulation if mentioned on a source with a higher weighting, which has not been accounted for in this study. Articles written in languages other than English were excluded. A comparison of the top-20 most cited studies and highest AAS may not be generalizable to other databases. In addition, this study lacks a statistical analysis.

## Conclusions

The top-20 studies according to AAS or citation count are not the same. The top-20 studies by AAS are composed of studies at higher levels of evidence versus the top-20 studies when listed by citation count.

## Disclosures

All authors (L.O., C.L., M.S.D., A.K., A.H., J.T.C.) declare that they have no known competing financial interests or personal relationships that could have appeared to influence the work reported in this paper.
